# A meta-analysis of interaction between Epstein-Barr virus and HLA-DRB1*1501 on risk of multiple sclerosis

**DOI:** 10.1038/srep18083

**Published:** 2015-12-11

**Authors:** Di Xiao, Xingguang Ye, Na Zhang, Meiling Ou, Congcong Guo, Baohuan Zhang, Yang Liu, Man Wang, Guang Yang, Chunxia Jing

**Affiliations:** 1 Department of Epidemiology, School of Medicine, Jinan University, Guangzhou, China; 2 Department of Parasitology, School of Medicine, Jinan University, No.601, Huangpu Avenue West, Guangzhou, Guangdong, China; 3 Key Laboratory of environmental exposure and health in Guangzhou, Jinan University, Guangzhou, Guangdong, China

## Abstract

Infection with Epstein-Barr virus (EBV) and HLA-DRB1*1501-positivity is a risk factor for multiple sclerosis (MS), but whether an interaction between these two factors causes MS is unclear. We therefore conducted a meta-analysis on the effect of the interaction between HLA-DRB1*1501 and EBV infection on MS. Searches of PubMed, Web of Science, China National Knowledge Infrastructure (CNKI), and the Wanfan databases through February 2015 yielded 5 studies that met the criteria for inclusion in the meta-analysis. EBV infection and HLA-DRB1*1501-positivity were dichotomized. The additive (S) and multiplicative interaction indexes (OR) between EBV infection and HLA-DRB1*1501 and their 95% confidence intervals (95%CI) were calculated for each study and then combined in a meta-analysis. EBV infection was significantly associated with MS (OR = 2.60; 95%CI, 1.48–4.59). HLA-DRB1*1501 was associated with a significantly increased risk of MS (OR, 3.06; 95%CI, 2.30–4.08). An interaction effect between EBV infection and HLA-DRB1*1501 on MS was observed on the additive scale (S, 1.43; 95%CI, 1.05–1.95, P = 0.023), but no interaction effect was observed on the multiplicative scale (OR, 0.86, 95%CI, 0.59–1.26). This meta-analysis provides strong evidence that EBV alone, HLA-DRB1*1501 alone or their interaction is associated with an elevated risks of MS.

Multiple sclerosis (MS) is a common chronic inflammatory demyelinating disease of the central nervous system (CNS) that causes severe progressive disability, particularly in young people, and affects more than 2.5 million people worldwide^1^. MS is a chronic immune-mediated disorder with a complex etiology. The pathogenesis of the disease is not well understood. Both environmental and genetic factors have been implicated in disease causation^2^. In a large genome-wide association study (GWAS), 465,434 autosomal single nucleotide polymorphisms (SNPs) were analyzed in 9772 cases and 17,376 controls of European descent; the strongest association was between HLA-DRB1*1501 and MS (OR = 3.1, P = 1 × 10^−320^)^3^. Furthermore, the HLA-DRB1*1501 allele is considered to be a definite, strong risk factor for MS^4^,^5^. Carriers of HLA-DRB1*1501 have an up to four-fold increased risk of MS^6^.

Serological data from prospective and retrospective studies suggest that past EBV infection is a prerequisite for MS development^7^,^8^,^9^. A cohort study of US military personnel has demonstrated that anti-EBNA-1 titers are associated with a 3-fold increased risk of MS^10^. In addition, a meta-analysis has revealed a significant OR for sero-positivity to anti-EBNA IgG in MS cases (4.5[95% confidence interval (CI) 3.3 to 6.6, p < 0.00001])^11^.

The presentation of viral or bacterial peptide antigens to CD4^+^ T cell receptors may induce cross-activation with self-antigens^12^. HLA-DRB1 may result in self-antigen cross-reactivity with EBV antigens in addition to serve as a co-receptor for EBV entry into B cells^13^,^14^,^15^. De Jager *et al*^16^ have suggested that HLA-DRB1*15 influences the CD4^+^ Th-mediated immune response to EBV infections^13^,^17^,^18^. In Individuals with HLA-DRB1*1501,EBV-infected B cells may present CNS self-antigens to CD4^+^ T cell receptors, thereby inducing an autoimmune response^19^.

Although the independent effects of EBV infection and HLA-DRB1*1501 on the risk of MS have been established^6^,^16^, the possible interaction between these factors is not well characterized. Moreover, data from individual studies on the interaction between HLA-DRB1*1501 and EBV infection are not entirely consistent. Some studies have report no interaction^16^,^20^,^21^. Whereas other studies have observed an interaction between EBV infection and HLA-DRB1*1501 in the risk of MS^22^,^23^. Thus, whether the interaction of two factors increases the risk of MS remains controversial.

To better illuminate the independent and combined effects of EBV infection and HLA-DRB1*1501 in the etiology of MS, we conducted a meta-analysis to evaluate the interaction between these two factors in MS risk.

## Results

### Result of the literature search

**Figure 1:**
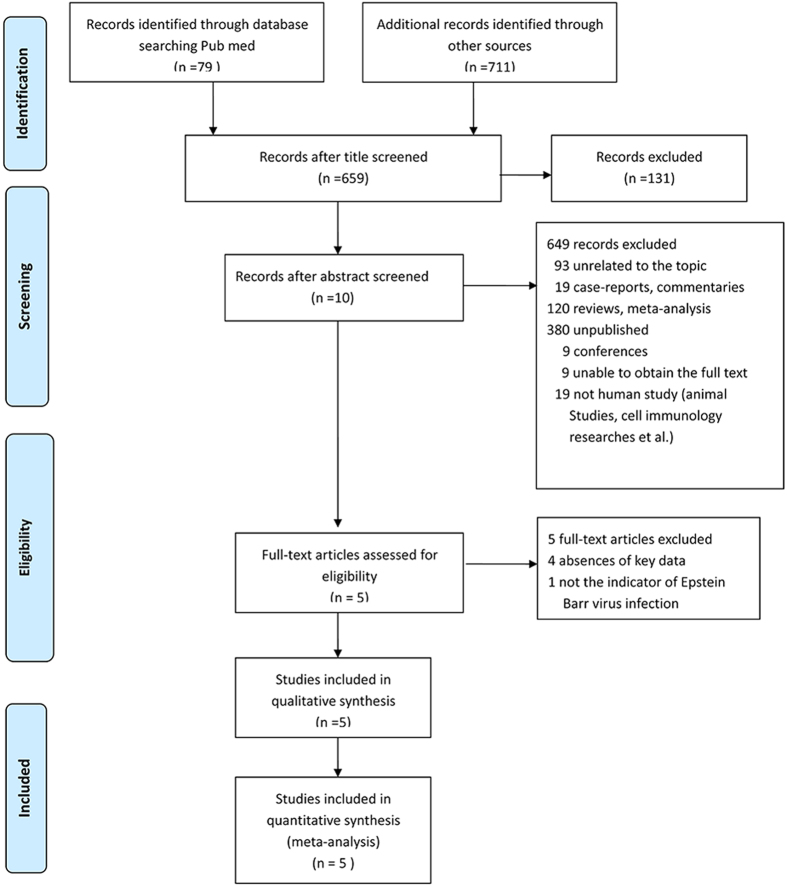
Flowchart of the meta-analysis of the interaction between HLA-DRB1*1501 and EBV infection on the risk of MS.

A total of 659 studies were identified from a primary literature search after 131 duplicates were excluded. A total of 648 of the 659 records were excluded:19 case-reports and commentaries, 92 studies irrelevant to the topic,19 non-human studies, 120 reviews and meta-analyses, 380 studies including unpublished data; 9 conference reports; and 9 reports for which full text was unavailable. The remaining 11 studies were scanned, and 6 additional studies were excluded for the following reasons: one study was not an indicator of EBV, and five lacked key data. Finally, 5 studies were considered eligible for this meta-analysis (Fig. 1).

The general characteristics of all 5 studies are presented in Table 1. HLA-DRB1*1501 was genotyped in three studies, and in van der Mei *et al.*, rs3135005 was genotyped as a proxy for the HLA-DRB1*1501 allele^20^. One study only distinguished HLA-DRB1*15 as HLA-DRB1*1501 without specific alleles. IgG antibodies to EBNA-1 were measured in all 5 studies as an indicator of EBV infection. The studies included a total of 2533 participants, including 1069 MS patients. One study was from India, one was from Canada, one was from Australia, and the remaining studies were from Sweden.

### Meta-analysis of the interaction between EBV infection and HLA-DRB1*1501

There was significant interaction between HLA-DRB1*1501 positivity and EBV infection based on the additive scale (S = 1.43, 95%CI = 1.05–1.95, P = 0.023; AP = 0.29, 95%CI, 0.12–0.47, P = 0.001; RERI = 1.44, 95%CI, 0.30–2.58, P = 0.013) (Table 2). HLA-DRB1*1501-positive individuals infected with the EB virus had a higher risk (OR = 6.11, 95%CI: 3.84–9.74) of MS.

**Figure 2:**
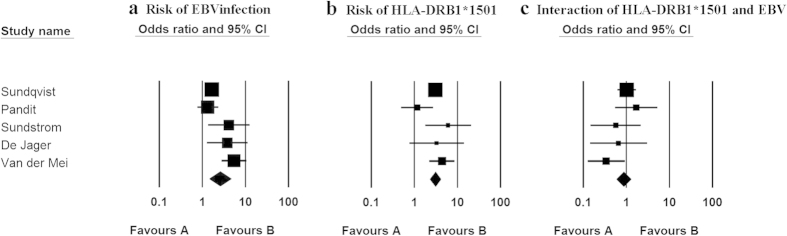
Logistic regression analyses of EBV infection and HLA-DRB1*1501 on risk of MS. The boxes and lines indicate the odds ratios (ORs) and their 95% confidence intervals (CIs) on a log scale for each study. The size of the box indicates the relative weight of each estimate.

Multiplicative interaction analysis revealed no interaction between EBV infection and HLA-DRB1*1501 (OR = 0.86, 95%CI = 0.59–1.26, P = 0.449) (Supplementary Table S2 online). However, EBV infection alone was significantly associated with MS (OR, 2.60; 95%CI, 1.48–4.59) (Fig. 2a), and HLA-DRB1*1501 significantly increased the risk of MS (OR, 3.06; 95%CI, 2.30–4.08) (Fig. 2b).

### Publication bias and heterogeneity

There was no publication bias for the interaction in either the additive model (Supplementary Figure S1 online) or multiplicative model (Supplementary Figure S2 online). The Egger test also did not detect any evidence of publication bias on the additive scale (AP: SE = 0.817, P = 0.581; RERI: SE = 0.590, P = 0.685; S: SE = 0.92, P = 0.807) or multiplicative scale (SE = 1.36, P = 0.526).

The heterogeneity test detected low heterogeneity across the additive scale (AP: I^2^ = 0.000, P = 0.833; RERI: I^2^ = 0.000, P = 0.824; S: I^2^ = 0.000, P = 0.723) and multiplicative scale (I^2^ = 31.758, P = 0.210). Both interaction scales were estimated by a fixed model.

In all 5 studies, the median number of items fulfilled on the STREGA and STROBE checklists was 17 (range 13 to 18). Furthermore, as shown in Supplementary Table S3 online, the criteria for evaluating the quality of this meta-analysis were clearly described for all included studies.

## Discussion

The interaction between HLA-DRB1*1501and EBV in MS remains unclear because of the conflicting results in existing studies. To address this issue, we conducted a meta-analysis of published studies. We identified a significant additive interaction between EBV infection and HLA-DRB1*1501 in the risk of MS; however, we did not observe an interaction based on the multiplicative scale.

For G^+^/E^+^ and G^+^/E^−^ exposures, there were more frequencies in the case group than control group (39.5% vs 17.6%, P = 0.000, in G^+^/E^+^; 12.4% vs 10.9%, P = 0.014, in G^+^/E^−^). 12.9% of cases and 33.9% controls were in G^−^/E^−^ with statistical difference (Z = −10.64, P = 0.000). Finally, there was no statistical difference (Z = −0.76, P = 0.445) for the frequency of G^−^/E^+^ in between cases (30.4%) and control (33.4%). These results suggested that both HLA-DRB1*1501 and EBV-positive contribute the occurrence of MS in the population. Especially, when the individuals carry the susceptible HLA-DRB1*1501gene, once are infected by EB virus, they have more risk to suffer from MS.

In our meta-analysis, the pooled S was 1.43 (95%CI, 1.05–1.95, P = 0.023); RERI was 1.44 (95%CI = 0.30–2.58, P = 0.013) and the AP was 0.29 (95%CI = 0.12–0.47, P = 0.001). All three indexes indicated that there was indeed a biological interaction between HLA-DRB1*1501 and EBV^24^. S also indicated that there was a significant synergistic interaction based on the additive scale.

HLA-DRB1*1501 was associated with a 3-fold elevation in MS risk, and EBV infection was associated with a 2.6-fold elevation in MS risk. Furthermore, our data indicate that the combined effects of HLA-DRB1*1501 positivity and Epstein-Barr virus infection result in an up to six-fold increased risk of MS. These findings showed us the importance of the interaction effects between HLA-DRB1*1501 and EBV infection on the occurrence of MS. How the interaction between these two factors contributes to the increased risk of MS remains unclear. One possible mechanism includes HLA class II molecules, which are involved in the processing and presentation of foreign antigens in the immune defense process; this process mainly occurs on the surface of antigen-presenting cells. Therefore, HLA-DRB1*1501 may interfere with this process and prevent the presentation of EBV antigens to CD4 ^+^  Th cell receptor, thereby inhibiting immune defense recognition^9^,^25^, and leading to EBV accumulation in B cells^26^. EBV-infected B cells distributed in the CNS can present CNS antigens, the molecular mimicry of EBV^13^,^27^,^28^, to CD4 ^+^  T-cell receptors under the influence of the virus and may activate cellular and humoral immune responses^26^,^29^. Dysfunctional immune regulation induces the excitation of autoimmune responses. Furthermore, EBV-infected B cells can provide costimulatory survival signals to T cells and protect activated CD4 ^+^ T-cell from elimination by immunoregulation, thereby leading to the development of MS^19^. In another aspect, in the development of the thymus, particular HLA molecules cannot very well present self-antigen to developing T cells to undergo effective negative selection process, which lead to those auto-reactive T cells persisting even after the individual mature and launching an immune attack against the self-antigen under certain conditions^30^,^31^. Therefore, when EBV infection makes B cells presenting self-antigens^13^,^27^,^28^, auto-reactive T cells can recognize these self-antigens, which further accelerate the progression of MS^30^. So, when individuals carrying special HLA genotype are infected by EBV, those two factors influence and promote each other, which accelerate the progression of MS.

Our meta-analysis was comprehensive because we assessed the interaction based on both additive and multiplicative scales. We chose this approach because both scales are informative, and arguments can be made in favor of each of the two scales^32^. Distinguishing gene-environment interactions that may reflect biologic processes such as molecular mimicry will contribute to further dissection of the disease mechanisms that culminate in MS onset^33^ and provide new insights for the treatment and prevention of MS. Our meta-analysis suggested that the risk of MS would be increased greater when HLA-DRB1*1501 and EBV occur together. Moreover, we evaluated the primary effects of HLA-DRB1*1501 and EBV in MS by performing an extensive analysis of the expectations of the gene-environment interaction.

This meta-analysis has important clinical implications. Due to hereditary nature of the HLA-DRB1*1501 genotype, effective intervention and prevention measures can be implemented by monitoring EBV infection status. An increasingly large body of evidence has indicated that EBV infection plays an essential role in the development of MS, raising the possibility that MS maybe prevented and potentially cured by controlling EBV infection^34^. Reducing the maximum exposure to EBV may significantly reduce the risk of MS. Consequently, we suggest that EBV infection should be prevented in healthy individuals (particularly HLA-DRB1*1501 carriers). Vaccination of healthy EBV-seronegative young adults with recombinant gp350 is effective in preventing the development of infectious mononucleosis induced by EBV infection, although it does not prevent asymptomatic EBV infection^35^. Healthy HLA-DRB1*1501-positive individuals should receive regular screening to measure serum titers of anti-EBNA antibodies^36^. Furthermore, the development and application of a vaccine against EBV may reduce the risk of MS. Our findings may have significance for the prevention of the occurrence or recurrence of MS. Moreover, the immune response to EBV may be a therapeutic option in MS^34^. In MS patients infected with EBV, MS may be cured by controlling the immune response to EBV infection. Some evidence supports a beneficial effect of vitamin D3 on reducing antibody titers against EBV in MS patients^37^. We currently prescribe rituximab to boost immunity to EBV and antiviral drugs to treat EBV infection^34^.

Some limitations of our study merit further discussion. Exposures other than the study exposures exhibited disequilibrium, particularly smoking. For example, in one study an interaction was observed on the multiplicative scale between EBNA1 IgG and smoking^21^. It is difficult to exclude a potential confounding effect of smoking because smoking is associated with many populations. Therefore, the so-called population imbalance stratification of other exposures may interfere with the results of this study. The case-control study in the meta-analysis may also introduce selection bias. Another limitation of our meta-analysis is that the method used to calculate the interaction on the additive scale might only apply to two factors at two levels. When the variable factors were multiply variable, 95% confidence intervals of S, AP and RERI were not calculated by the Excel calculation spreadsheet used in this study. The 5 independent studies were from different countries with the different genotyping methods for HLA-DRB1*1501. Although these methods were different, they are in consensus at genotyping HLA-DRB1*1501. Hence, the results would not be influenced by the differences in the genotyping methods for HLA-DRB1*1501. Therefore, results are reliable.

Most of the included studies were Caucasian samples and only one study was India population, which might influenced the results. We failed to identify a significant interaction between HLA-DRB1*1501 and Epstein-Barr virus on the risk of MS in the population from India. This result may have two explanations. First, compared to the other included studies, the Indian study had a restricted sample size, which may have limited the power to evaluate the interaction. Therefore, large-scale studies are needed to validate the interaction between HLA-DRB1*1501 and Epstein-Barr virus in MS in Indian populations. Second, ethnic differences may underlie this result. As the prevalence of 3/100,000 in MS was low in India, while the prevalence varies between 60 and 200 per 100,000 in people of north American and northern European origin^2^. It is possible that the interaction between HLA-DRB1*1501 and Epstein Barr virus in MS is relevant to only Caucasian populations and not other ethnic groups. Further studies are needed to test the interaction between HLA-DRB1*1501 and Epstein Barr virus in MS among other ethnic groups by recruiting patients with non-Caucasian backgrounds^38^.

In conclusion, our meta-analysis identified an interaction between HLA-DRB1*1501 and EBV infection for the risk of MS on an additive scale; however, we did not observe a significant interaction between these factors on a multiplicative scale. Further study is needed to assess the direct evidence and understand the potential mechanism underlying this finding.

## Methods

### Study identification

A search of the Pubmed , Web of Science, CNKI and the Wanfang databases was conducted through February 2015 using the search terms HLA, multiple sclerosis, Epstein Barr virus, and interaction.

The retrieved studies fulfilled the following criteria for inclusion in the meta-analysis: (a) multiple sclerosis, (b) EBV infection (EBNA-1 IgG as the index of EBV infection), (c) genotyped HLA-DRBA*1501 status (any method) and (d) the interaction of EBV infection and HLA-DRB1*1501 in MS. Studies were excluded from our analysis if there was an absence of detailed numbers in one of the following four groups: HLA-DRB1*1501-negative subjects without EBV infection; HLA-DRB1*1501-positive subjects with EBV infection; HLA-DRB1*1501-negative subjects with EBV infection; and HLA-DRB1*1501-positive subjects without EBV infection.

### Data extraction

Data were independently extracted by two investigators (Di Xiao and Xingguang Ye) who were blinded to each other, using a data recording developed for this purpose. When detailed data were lacking, we attempted to contact the corresponding author to obtain the original data. Studies were excluded if the authors did not provide additional data. Any disagreement between the two data extractors was resolved by consensus. After extraction, the data were also reviewed and compared by Chunxia Jing.

### Quality assessment

All included studies were assessed based on the STREGA (Strengthening the Reporting of Genetic Association Studies) and STROBE (Strengthening the Reporting of Observational Studies in Epidemiology) checklists^39^,^40^.

The assessment involved six domains, including title and abstract, introduction, methods, results, discussion and other information. Each item was classified with “ + ” or “−”, which represented fulfillment of the checklist criteria or a lack of fulfilment of the criteria, respectively.

### Statistical Analysis

The serum level of IgG antibodies against the Epstein-Barr virus (EBV) nuclear antigen 1 (EBNA-1) is a strong risk factor for MS^16^,^41^,^42^. Because antibodies to the EBNA-1 antigen have emerged as the most consistent predictor of MS in multiple serological studies^9^,^10^,^43^, the anti-EBNA-1 titer was used as the index of the immune response to EBV infection in our meta-analysis.

In all the included studies, the EBNA-1 antibody titer was used as an index of EBV infection rather than EBNA-2(another individual component of the EBNA family), the EBV viral capsid antigen (VCA) or the anti-early antigen complex (diffuse [EA-D]).

Our stratification differed from some original studies^2^,^6^,^16^. For example, two studies^6^,^16^ used three types of EBNA-1 antibody titers (low/medium/high), which were converted into a dichotomous variable in our meta-analysis. The high IgG level used in this meta-analysis was combined with the medium and high IgG titers in the original studies. One study^2^ used an inter-quartile stratification, and we used the value above the 50th percentile of the inter-quartile range as the high IgG level. Low EBNA-1 antibody titers were defined as non-EBV infection, and high EBNA-1 antibody titers were defined as EBV infection.

HLA-DRB1*1501 was also considered a dichotomous variable (positive/negative), consistent with all of the original studies.

The interaction effects were determined by using two models: logistic regression to assess the interaction on the multiplicative scale, and S (the synergy index), RERI (the excess risk due to interaction) and AP (the attributable proportion due to interaction) to assess the interaction on the additive scale.

In additive model, we categorized the study subjects into four groups according to HLA-DRB1*1501 and EBV infection status: HLA-DRB1*1501-negative and EBV-negative (RR_00_), HLA-DRB1*1501-positive and EBV-negative (RR_10_), HLA-DRB1*1501-negative and EBV-positive (RR_01_), and HLA-DRB1*1501-positive and EBV-positive (RR_11_). We defined subjects who were unexposed to both risk factors as the reference category (i.e., RR_00_ = 1). These relative risk estimates can be obtained from a logistic regression model. The corresponding covariance matrix and regression coefficients are also needed to calculate the confidence intervals^24^. To obtain adequate estimates, the model was established with indicator variables for each of the four different combinations of exposure. For convenience, we structured a new variable C and defined it as three indicator variables: dum01, dum10 and dum11 (see Supplementary Table S1 online). An Excel spreadsheet (www.epinet.se) was used to calculate additive interaction: S, RERI and AP. AP refers to the attributable proportion of disease that is due to interaction among individuals with both exposures. S is the excess risk from both exposures when there is an additive interaction, relative to the risk from both exposures without interaction. RERI >0, AP > 0, or S > 1 indicates biological interaction^24^,^44^. Furthermore, S > 1 for synergetic effects and S < 1 for antagonistic effects^24^,^45^,^46^.

In the multiplicative model, we fit a multiple logistic regression model with the response variable MS (case/control) and independent variables HLA-DRB1*1501(G), EBV infection (E), and their product G × E. The odds ratio of G × E was the index of the multiplicative model interaction between HLA-DRB1*1501 and EBV infection in the risk of MS. A multiplicative interaction existed when the 95% confidence intervals of OR did not contain 1. Otherwise, the result was reversed.

Sensitivity analysis was performed by removing one study at a time to assess whether the meta-estimates were strongly influenced by any individual study. We used the forest plot and Egger’s regression intercept to assess publication bias^47^,^48^.The following cutoffs were used to evaluate heterogeneity: I^2^ = 0–25%, no heterogeneity; I^2^ = 25–50%, moderate heterogeneity; I^2^ = 50–75%, large heterogeneity; and I^2^ = 75–100%, extreme heterogeneity^49^. Pooled mean differences were estimated by using a fixed-effects model when there was no heterogeneity or moderate heterogeneity (I^2^ < 50%) and a random-effects model when there was moderate, large, or extreme heterogeneity (I^2^ ≥ 50%)^50^. All statistical analyses were conducted using SPSS version 16.0(SPSS Inc, Chicago, USA), Microsoft Excel2007 (Microsoft, Redmond, WA, USA), and Comprehensive Meta Analysis V2 (Biostat Inc, USA).

## Additional Information

**How to cite this article**: Xiao, D. *et al.* A meta-analysis of interaction between Epstein-Barr virus and HLA-DRB1*1501 on risk of multiple sclerosis. *Sci. Rep.* **5**, 18083; doi: 10.1038/srep18083 (2015).

## Supplementary Material

Supplementary Information

## Figures and Tables

**Table 1:** Summary characteristics of the studies selected.

Author	Year	Gene locus	EBV infection indicator	Country	STREGA and STROBE checklist (total = 22)	Total No. of Participants	G^+^/E^+^				G^+^/E^−^				G^−^/E^+^				G^−^/E^−^			
							case	(%)	control	(%)	case	(%)	control	(%)	case	(%)	control	(%)	case	(%)	control	(%)
Sundqvist^42^	2012	HLA-DRB1* 15	EBNA-1 IgG	Sweden	17	1177	213	38.6	101	16.2	113	20.5	92	14.7	136	24.6	206	33.0	90	16.3	226	36.2
Pandit^2^	2013	HLA-DRB1* 1501	EBNA-1 IgG	India	15	280	32	22.4	15	10.9	13	9.1	14	10.2	48	33.6	45	32.8	50	35.0	63	46.0
Sundstrom^6^	2008	HLA-DRB1* 1501	EBNA-1 IgG	Sweden	13	321	60	55.0	49	23.1	13	11.9	25	11.8	32	29.4	91	42.9	4	3.7	47	22.2
De Jager^16^	2008	rs3135005replace HLA-DRB1* 1501	EBNA-1 IgG	Canada	18	416	71	50.7	79	28.6	5	3.6	14	5.1	60	42.9	146	52.9	4	2.9	37	13.4
Van der Mei^20^	2010	rs3135005replace HLA-DRB1* 1501	EBNA-1 IgG	Australia	18	339	42	33.6	25	11.7	26	20.8	29	13.6	30	24.0	27	12.6	27	21.6	133	62.1

EBV, Epstein-Barr virus; EBNA-1, Epstein-Barr virus nuclear antigen 1; IgG, immunoglobulin; G^+^, HLA-DRB1*1501/HLA-DRB1*15 positive; E^+^, EBV positive; G^−^, HLA-DRB1*1501/HLA-DRB1*15 negative; E^−^, EBV negative; OR, odds ratio; CI, confidence interval.

**Table 2:** The interaction of risk estimates between HLA-DRB1*1501 and EBV based on the additive scale.

Author	RERI	95%CI for RERI	P	AP	95%CI for AP	P	S	95%CI for S	P
Sundqvist^42^	1.55	−0.01–3.11	0.051	0.29	0.05–0.54	0.019a	1.57	0.99–2.49	0.053
Pandit^2^	1.17	−0.78–3.12	0.238	0.44	−0.10–0.97	0.110	3.28	0.26–42.35	0.362
Sundstrom^6^	5.15	−3.04–13.33	0.218	0.36	−0.04–0.76	0.078	1.62	0.81–3.25	0.171
De Jager^16^	2.21	−2.41–6.83	0.349	0.27	−0.24–0.77	0.301	1.43	0.63–3.29	0.395
Van der Mei^20^	−0.61	−6.16–4.94	0.828	−0.07	−0.77–0.62	0.835	0.92	0.44–1.92	0.828
pooled	1.44	0.30–2.58	0.013^a^	0.29	0.12–0.47	0.001^a^	1.43	1.05–1.95	0.023^a^

RECI, excess risk due to interaction; AP, attributable proportion due to interaction; S, the synergy index; Cl, confidence interval.

^a^Statistically significant (P < 0.05).
