# Supplementary material for: A meta-analysis of interaction between Epstein-Barr virus and HLA-DRB1*1501 on risk of multiple sclerosis
Source: Sci Rep. 2015 Dec 11;5:18083. doi: 10.1038/srep18083 (PMC4676020; doi:10.1038/srep18083)
Supplement: Supplementary Information [file srep18083-s1.doc]

A meta-analysis of interaction between Epstein-Barr virus and HLA-DRB1*1501 on risk of multiple sclerosis

Di Xiao, Xingguang Ye, Na. Zhang, Meiling Ou, Congcong Guo, Baohuan Zhang, Yang Liu, Man Wang, Guang Yang & Chunxia Jing

**Supplementary information**

**Table S1.** Definition of dummy variables for different exposure combinations.

| HLA-DRB1*1501 | EBV | C | Dum10 | Dum01 | Dum11 | OR |
| --- | --- | --- | --- | --- | --- | --- |
| 0 | 0 | 0 | 0 | 0 | 0 | OR_00_ |
| 1 | 0 | 1 | 1 | 0 | 0 | OR_10_ |
| 0 | 1 | 2 | 0 | 1 | 0 | OR_01_ |
| 1 | 1 | 3 | 0 | 0 | 1 | OR_11_ |

EBV, Epstein-Barr virus; OR, odds ratio; CI, confidence interval.

**Table S2. The interaction of risk estimates and 95% confidence intervals between**

HLA-DRB1*1501 and Epstein-Barr virus on multiplicative scale.

| Author | OR | 95%CI | P |
| --- | --- | --- | --- |
| Sundqvist[^42^](#_ENREF_42) | 1.04 | 0.64-1.69 | 0.087 |
| Pandit[^2^](#_ENREF_2) | 1.71 | 0.56-5.23 | 0.347 |
| Sundstrom[^6^](#_ENREF_6) | 0.57 | 0.15-2.18 | 0.411 |
| De Jager[^16^](#_ENREF_16) | 0.66 | 0.15-3.02 | 0.594 |
| Van der Mei[^20^](#_ENREF_20) | 0.34 | 0.13-0.92 | 0.032^a^ |
| pooled | 0.86 | 0.59-1.26 | 0.449 |

OR, odds ratio; CI, confidence interval. ^a^Statistically significant (P0.05).

| **Table S3. Quality assessment of HLA-DRB1*1501, Epstein-Barr virus and MS studies.** | | | | | |
| --- | --- | --- | --- | --- | --- |
| **Study** | Sundqvist[^36^](#_ENREF_36) | Pandit[^2^](#_ENREF_2) | Sundstrom[^6^](#_ENREF_6) | De Jager[^16^](#_ENREF_16) | Van der Mei[^20^](#_ENREF_20) |
| **Title&Abstract** |  |  |  |  |  |
| Informative and balanced summary | ＋ | ＋ | ＋ | ＋ | ＋ |
| **Introduction** |  |  |  |  |  |
| Background& rationale clear reported | ＋ | ＋ | ＋ | ＋ | ＋ |
| Objectives& hypothesis clear stated | ＋ | ＋ | ＋ | ＋ | ＋ |
| **Method** |  |  |  |  |  |
| Key elements of study design presented | ＋ | ＋ | ＋ | ＋ | ＋ |
| Sufficient descriptive setting, location and relevant date | ＋ | ＋ | ＋ | ＋ | ＋ |
| Clear eligibility criteria for case studies &matching criteria for matched studies | ＋ | ＋ | － | ＋ | ＋ |
| Clear definition for each variable | ＋ | ＋ | － | ＋ | ＋ |
| Data sources measurement clear presented | ＋ | ＋ | － | ＋ | ＋ |
| Assessment of bias | － | － | － | － | － |
| Date size explained | － | － | － | － | － |
| Assessment of quantitative variables | － | ＋ | － | ＋ | ＋ |
| Statistical methods replicable | ＋ | ＋ | ＋ | ＋ | ＋ |
| **Results** |  |  |  |  |  |
| Clear descriptive participants at each stage | ＋ | ＋ | ＋ | ＋ | ＋ |
| Sufficient descriptive data (eg. Demographic) | ＋ | － | － | ＋ | ＋ |
| Clear outcome data | ＋ | ＋ | ＋ | ＋ | ＋ |
| Estimate of result clear stated | ＋ | ＋ | ＋ | ＋ | ＋ |
| Other analysis stated (eg. Subgroup analysis ,sensitivity analysis) | ＋ | － | ＋ | ＋ | ＋ |
| **Discussion** |  |  |  |  |  |
| Key results clear summarized | ＋ | － | ＋ | ＋ | ＋ |
| Limitation clear stated | ＋ | － | － | － | － |
| Cautious overall interpretation | ＋ | ＋ | ＋ | ＋ | ＋ |
| Generalizability stated | － | － | － | － | ＋ |
| **Other information** |  |  |  |  |  |
| Source of funding stated | － | ＋ | ＋ | ＋ | － |


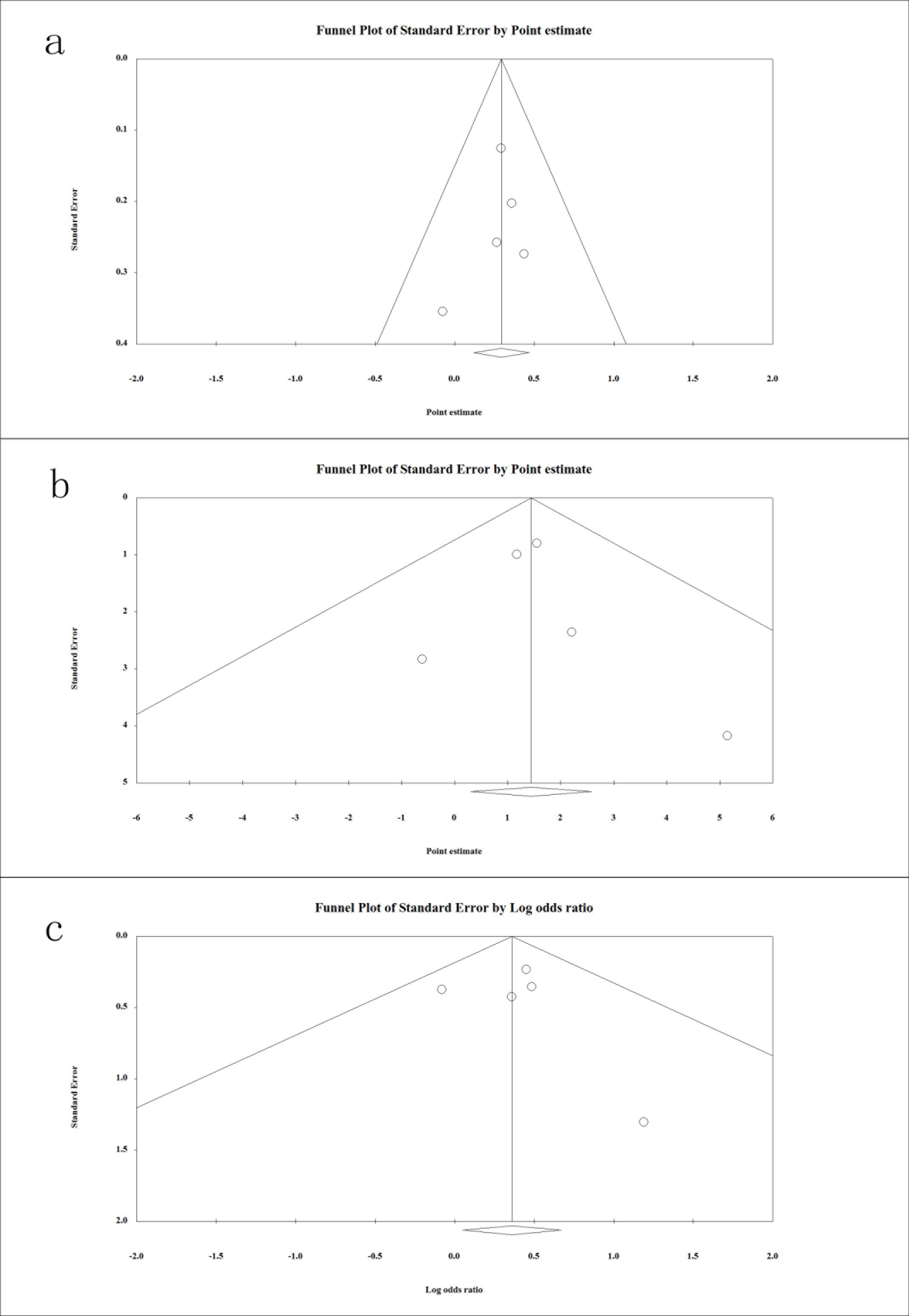


**Figure S1．Funnel plots for publication bias in the additive interaction between HLA-DRB1*1501 and EBV infection on the risk of MS (a: AP; b: RERI; c: S).**


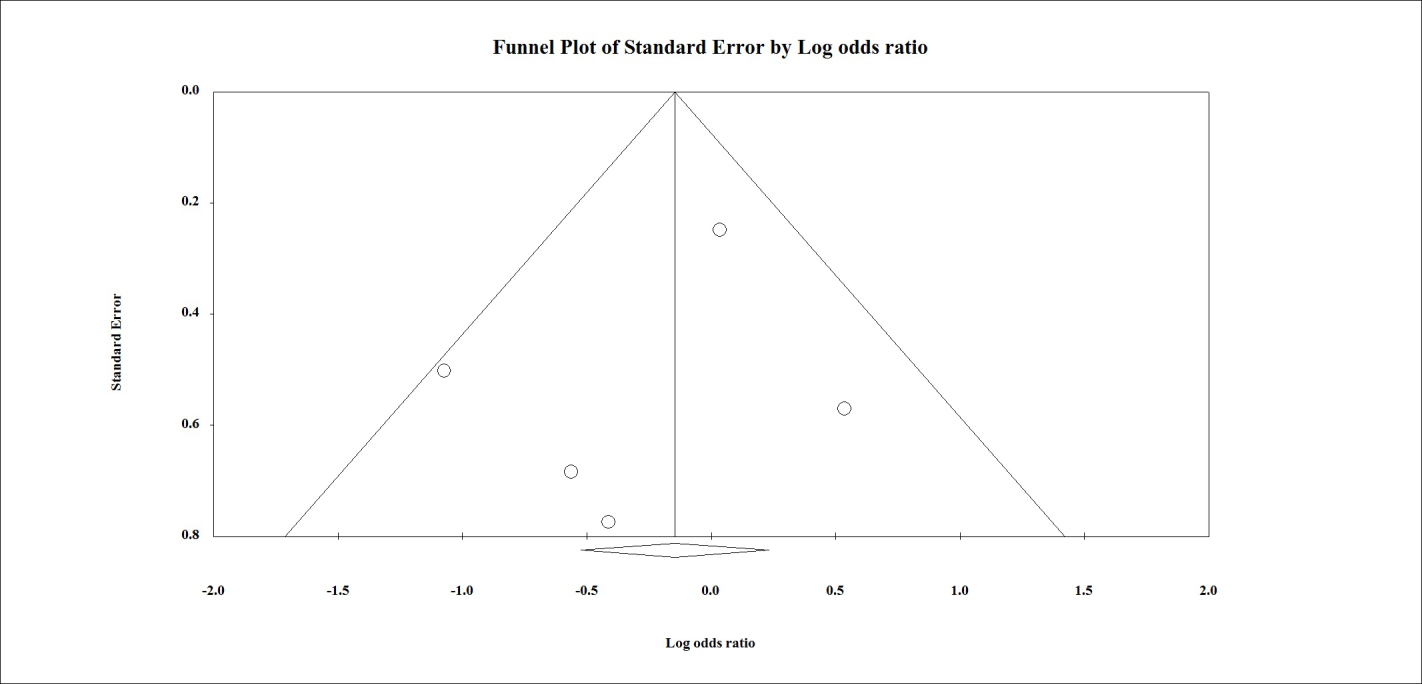


**Figure S2．Funnel plots for publication bias in the multiplicative interaction between HLA-DRB1*1501 and EBV infection on the risk of MS.**
